# The risk for nonpsychotic postpartum mood and anxiety disorders during the COVID-19 pandemic

**DOI:** 10.1177/0091217420981533

**Published:** 2020-12-15

**Authors:** Jelena Stojanov, Miodrag Stankovic, Olivera Zikic, Matija Stankovic, Aleksandar Stojanov

**Affiliations:** 1 Special Hospital for Psychiatric Disorders, Gornja Toponica, Serbia; 2 Faculty of Medicine, University of Nis, Nis, Serbia; 3 Center of Mental Health Protection, Clinical Centre Nis, Nis, Serbia; 4 The Mahindra United World College, Pune, India; 5 Clinic of Neurology, Clinical Center Nis, Nis, Serbia

**Keywords:** COVID-19 pandemic, postpartum, mood, anxiety, mental disorders

## Abstract

**Objective:**

The coronavirus disease 2019 (COVID-19) appears to be the largest pandemic of our times. The aim was to recognize the risk factors for nonpsychotic postpartum mood and anxiety disorders (NPMADs) in women during the pandemic and state of emergency police lockdown in Serbia.

**Methods:**

We assessed 108 postpartum women who completed the Edinburgh Postnatal Depression Scale (EPDS) and an additional survey constructed for this study. We also used the additional, previously mentioned survey, in 67 healthy age-matched women with children who were ≥2 years of age. The additional survey allowed us to gain insight into the impact of the pandemic as well as postpartum period on the risk of NPMADs.

**Results:**

In 16 (14.8%) subjects we found a score ≥10 on EPDS. Higher rates on the EPDS were noticed in elderly, single, and unemployed, women who lost their jobs due to the pandemic, or women who were dissatisfied with their household income (p < 0.05). The risk of NPMADs was linked significantly to quarantine, and social isolation, the absence of social support, as well as having emotional problems. Postpartum women, compared to non-postpartum women, were more anxious and had feelings of helplessness during social isolation.

**Conclusion:**

Understanding the factors that increase the risk of NPMADs during the pandemic could help prevent mental disorders during a possible future pandemic.

## Introduction

The coronavirus disease 2019 (COVID-19) appears to be the largest pandemic of our times, with no effective treatment available as of yet.^1^ Since the state of emergency was declared on March 15, 2020, and a police lockdown on March 18, 2020, Serbian media prioritized patient care, isolation, and reducing person-to-person transmission by insisting on a “Stay at Home period,” while restricting social interaction.^2^ Unethical media-related misinformation about COVID-19 is present in Serbia, as well as in other countries, resulting in fear/stress related to the unknown illness of this pandemic.^3^

Recent literature states that most health workers in hospitals are not trained to provide mental health assistance during COVID-19, and that psychological online assistance services by local or national mental health institutions overlooked the population’s maladaptive psychological reactions and emotional distress.^4–6^ Maladaptive psychological reactions and emotional distress were particularly neglected among those most severely affected by this pandemic, such as postpartum women.^7^

At a time of global crisis, fear, anxiety, and stress levels increase, with common stress responses, such as insomnia, health-anxiety, frustration, fear of being infected, and loneliness.^8^ The effects of protracted social isolation, massive closure of stores, layoffs – especially in the private sector – and a drastic reduction in the household budget, seem to impair functioning.^9^ Previous studies have reported high rates of anxiety, insomnia, depression, and stress symptoms among health care workers and patients infected or suspected of being infected, some studies showed the impact of COVID-19 in pregnant women, and in the early postpartum period, but none of them showed the impact on a wider range of one-year postpartum period thus far.^10–15^ The postpartum period is innately stressful, which is why postpartum women might be at an increased risk for mental disorders, due to COVID-19.^16^

Nonpsychotic postpartum mood and anxiety disorders (NPMADs) are, in current practice, defined by syndrome criteria, but also heavily rely on a clinician’s assessment and results of screening scales.^17^ Although NPMADs are among the most common morbidities in women of reproductive age, occurring in more than 10% of postpartum women, they remain at risk of being undiagnosed, underdiagnosed, and untreated.^18^ Previous knowledge points us to the possibility that NPMADs may result when postpartum women are unable to successfully adjust to a stressor – such as the COVID-19 pandemic – with longer mental health implications than the pandemic itself.^19^ Under the influence of a strong stressor, mothers with NPMADs show fewer positive caregiving behaviours, along with more exaggerated behaviours, with increased arousal and a tendency to withdraw when interacting with their children. This decreases the quality of the mother–child interaction.^20^

So far, Serbia has not successfully organized a system for the mental health-care of postpartum women at the state level. Before the pandemic appeared in Serbia (March 2020), we were studying the postpartum mental health of women, which gave us a unique opportunity to conduct this study. We applied the established framework and research methods to investigate the possible impact of COVID-19 on postpartum women.

The aim was to recognize the risk factors for NPMADs in women during the COVID-19 pandemic and state of emergency police lockdown in Serbia.

## Methods

We performed a cross-sectional study via an online survey over 7 days (from March 29th to April 4th), during the third week of Serbia’s state of emergency and police lockdown.

A total of 108 postpartum women (with children no older than 12 months) finished the online survey. Women included in the study are from the primary care parenting school database, and have been, at that time, a part of our research for a year; since the end of their third trimester of pregnancy. Excluding factors at the very start was the existence of antepartum mood or anxiety disorders, intense stress and trauma during pregnancy, the existence of postpartum mood, or anxiety disorders in the family, presence of chronic and autoimmune diseases, and drug therapy during pregnancy. We also assessed 67 age matched women with children that are ≥2 years of age, which served as the control group.

The survey form is composed of the Edinburgh Postnatal Depression Scale (EPDS), which is validated and standardized in Serbia, and additional questions concerning the relevant characteristics, reviewed from previous studies on postpartum women, constructed for this study.^21–23^ EPDS was given only to the group of postpartum women. EPDS is a reliable and valid screening tool for the detection of risks for postpartum depression, anxiety, and the entirety of NPMADs spectrum.^24,25^ It consists of ten self-reported items, in which, if the total score is ≥10, there is a risk of NPMADs.^21,24^ In our study, women were categorized into two groups: 1. At the risk of NPMADs (EPDS ≥10) and 2. Without risk of NPMADs (EPDS <10). They were compared with relevant characteristics.

Additional questions were divided into four sections: sociodemographic characteristics, mother and child characteristics, mother physical and mental health assessment, and mother’s perception of support. We collected the data regarding the age, place of residence, current partner status, level of the mothers’ and their partners’ education and employment status, terms of household income, type of delivery, parity and baby’s sex, perceptions of the overall satisfaction with support, as well as the overall importance of support. Additional questions about the physical and mental health assessment that we would normally ask during the interview were supplemented to form a more complete picture, given the limitations of EPDS.^25,26^ The survey form contained 42 questions in the Serbian language. This questionnaire was completed by all participants.

The study began by uploading the electronic version of the survey to the participants’ e-mails with a brief explanation of the survey and its purpose. Clicking the link automatically opened the survey. They were allowed to terminate the survey at any time.

The study procedures were carried out with the approval of the clinical research ethics committee of the Faculty of Medicine in Nis. All procedures were performed per the committee’s guidelines and regulations, including the Basics of Good Clinical Practice, the Declaration of Helsinki and the Law on Health Care of the Republic of Serbia. Verbal informed consent was provided before their enrolment, by phone, after which they were given information regarding the survey. All women stated that it would be more comfortable for them to participate if they remain anonymous. Hence, the survey was anonymous, provided online, and the confidentiality of information was assured. All participants provided informed consent via the online system.

All data were statistically processed by the IBM SPSS statistical software (version 21) for the Windows operative system. The research results are presented in tabular and graphic form. P values of less than 0.05 were regarded as statistically significant. Numerical data are presented as medians and interquartile range (IQR) for nonparametric data and as mean ± standard deviation (SD) for parametric data. The Mann-Whitney test was used to compare continuous variables between two groups, and the Kruskal-Wallis test was used to compare more than two groups. Correlations were assessed using Pearson’s correlation coefficients or Spearman’s correlation coefficients.

## Results

From a total of 108 women risk for NPMADs (score on EPDS ≥10) was found in 16 (14.8%), as opposed to 92 (85.2%), where no risk was found for NPMADs (score on EPDS <10).

### Socio-demographic characteristics

Sociodemographic characteristics of postpartum women are presented in Table 1. Higher rates on the EPDS were noticed in elderly (>35 years old), single, and unemployed, women who lost their jobs due to the pandemic, or women who were dissatisfied with their household income (p < 0.05). No statistically significant influence of their partners’ employment status, level of the participant’s education, their partner’s level of education, or place of residence was noted. Also we haven’t found a statistically significant correlation between scores obtained on EPDS with type of the delivery, number, and sex of the child.

**Table 1.** Sociodemographic characteristics of postpartal women, partners they live with and newborns and correlation with scores obtained on Edinburgh Postnatal Depression Scale (N = 108).

Variable	Value
Age* (median (min–max))	32 (19–45)
Urban area of living (%)	72.0
Currently unemployed* (%)	24.1
Lost job due to pandemic*(%)	13.9
Unsatisfied with incomes*(%)	19.5
Education (without university degree) (%)	29.1
Partner status (single)* (%)	13.0
Partner unemployed (%)	27.7
Highly educated partner (%)	62.7
Number of children (2 or more) (%)	14.8
Child sex (male) (%)	54.6
Natural delivery (%)	61.2

*p < 0.05.

### Mother physical and mental health assessment

**Figure 1.**
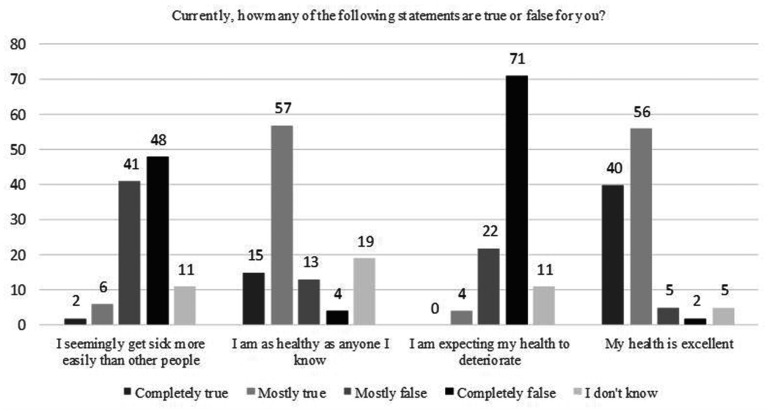
Expectations regarding impairment of physical health.

**Figure 2.**
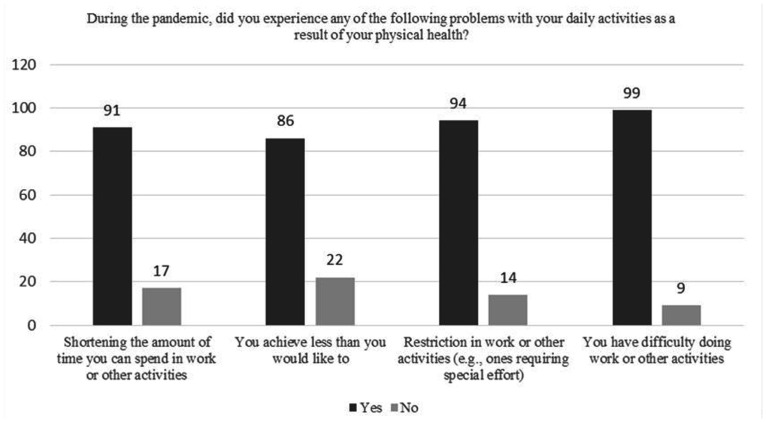
Physical health impact on daily functioning.

In regards to the questions about their current health, 99 (91.5%) of postpartum women describe their current health as good, while 100 (92.5%) estimate their health to be about the same as before the pandemic and state of emergency. Expectations of subjects regarding impairment of their physical health are presented in Figure 1. A physical health impact on daily functioning during the pandemic and state of emergency is shown in Figure 2. Reduced daily activities and achieving less than they wanted, as consequences of quarantine, also had a positive correlation with higher scores on EPDS (p < 0.01).

**Figure 3.**
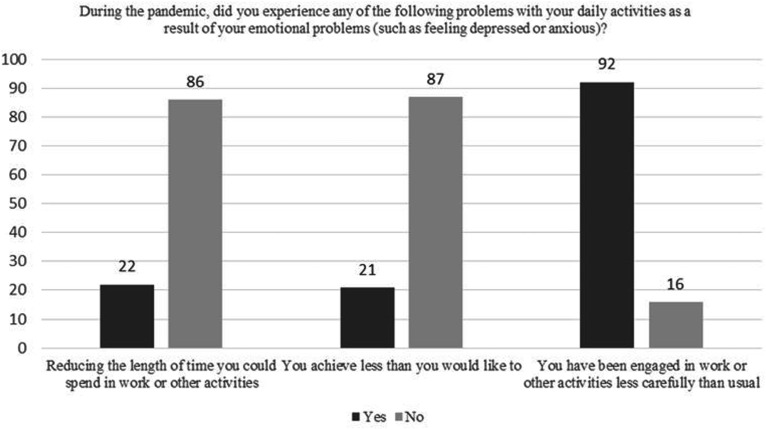
The impact of emotional problems on daily functioning.

**Figure 4.**
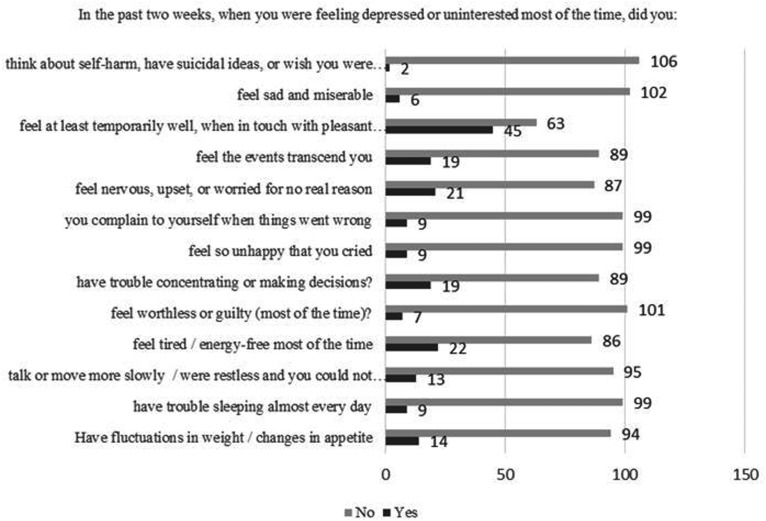
Additional symptomatology at times when they were feeling depressed or uninterested most of the time.

The impact of emotional problems on daily functioning during the pandemic and state of emergency is shown in Figure 3. There was a significant positive correlation in risk for NPMADs with working and achieving less, impacted by emotional problems (p < 0.05). Additional symptomatology at times when they were feeling depressed or uninterested most of the time is shown in Figure 4.

Only 2 subjects (1.9%) reported the exclusive impact and consequence of the pandemic and the state of emergency on their health, 20 (18.5%) stated that it was mostly related, 47 (43.5%), don’t see any association, and 39 (36.1%) estimated that “it is largely unrelated to it.” Regarding professional psychological help during the pandemic, 72 (66.7%) thought they do not need any professional help, 29 (26.8%) thought that professional support can help them to feel better, and 7 (6.5%) thought they need professional help, but they will not ask for it, because this will not be approved by their friends and family members.

### Mother’s perceptions of social support

During the pandemic and the state of emergency, 97 (89.8%) weren’t offered any assistance in supplying basic care items, as opposed to 11 (10.2%) who were offered assistance by family members. In 106 (98.1%) cases, women have not had any type of support, and 2 (1.9%) have had support from the family; 19 (17.6%) felt that it was important for them to have support and contact with services during the pandemic and the state of emergency, regarding the necessary information relating to protecting the health and meeting their own and their family’s needs. Higher rates on EPDS were noticed in the absence of social support (p < 0.01).

### Difference between postpartum women and non-postpartum women

We compared women who were in postpartum period and non-postpartum women. Considering their family’s income during the pandemic, postpartum women have experienced not having enough money and needing to borrow (p < 0.05). Postpartum women were more nervous, anxious or worried for no real reason, and have had negative feelings of helplessness (p < 0.01). They estimated no need for professional psychological support, absence of social support, and need for social support (p < 0.05).

## Discussion

Our results show that 14.8% of postpartum women have a risk of NPMADs during the third week of the pandemic and the state of emergency police lockdown in Serbia. The rate of a diagnosed mental disorder rise in the postpartum period and early identification of risk factors is important.^27^

Pandemics are severe stressors to vulnerable groups.^19,28^ In our sample, risk of NPMADs was associated with higher age, being unemployed and being single, which was present before the pandemic. Additionally, it has been confirmed in other studies.^29^

We have also found an association with certain uncommon consequences of a global catastrophe, such as being fired and dissatisfied with household income.^30^ Postpartum women, compared to non-postpartum women, were more dissatisfied with household income. They had the experience of not having enough money and needing to borrow, as a consequence of worrying and expecting a bad outcome of the pandemic.

We found no significance with characteristics such as partner’s employment status, level of education or place of residence, type of the delivery, and number and sex of the children. Although in previous studies, the risk of NPMADs was associated with these characteristics, it is possible that during the pandemic, they are not crucial and prominent.^30^

There is data on the impact of pandemics on postpartum women’s physical health, also mental health during pregnancy and in the early postpartum period, however, to our knowledge, there is very little information on their mental health on a wider range of one year postpartum period, during pandemics.^12–15,31,32^ In our study, more than 90% had a good current health assessment, and about the same as before the pandemic and the state of emergency. It differed from the assessment of physical and mental health.

The impediment of daily activities, that the quarantine and social isolation resulted in, significantly impairs physical and mental health, while increasing the risk for NPMADs. This was also observed in the other study groups.^1^ In our study, all women had sleeping problems, fluctuations in weight/changes in appetite, loss of energy, changes in speech and movement speed, problems with concentration, and had suicidal ideation. Postpartum women were more nervous, anxious, or worried for no real reason, and had negative feelings of helplessness. Less than 50% of all women could feel at least temporarily well, when in touch with pleasant content.

Protective apparels and social isolation were used to mitigate the risk of infection in the previous infectious outbreaks, such as the one in 2003, however, they included providing support.^33^ It is well known that one's perception or report of the support has a positive effect on the level of psychological stress that a person suffers. In this regard, help could alleviate the stress caused by the pandemic. It appears that one's perception or report of the support may act as a mediator between pandemic and stress, and is further related to the mental health of postpartum women.^34^

Psychological and social support in times of crisis is a protective factor for mental disorders in postpartum period.^35^ Our results show that postpartum women did not have any perception or report of the social support, although, it was important for them to have, or contact with services during the pandemic and state of emergency, while few felt they needed psychological support. Postpartum women compared to non-postpartum women perhaps overestimate the need for social support, and underestimate the need for psychological support, because of the postpartum period itself. Psychological perception of support was associated with psychological instability and the image they have of themselves; as a mother. Social perception of support in a quarantined situation and social isolation are probably more acceptable sources of support for them.

The analysis also showed that postpartum women who received some support from family were less depressed.^36^ The family system could help family members to adapt to the stress, through interpersonal relationships. In our sample only 10.2% of all women were offered, and 1.9% had family support, perhaps this is because pandemics such as COVID-19 could thrust a family into a state of instability, reduce the support that family members can give, or lead to family dysfunction. Therefore, it is necessary to organize support services for women in the postpartum period.^37^

Although the current study presents many innovative findings in our country, the results should be evaluated in the context of several limitations. First of all, we had a relatively small sample. They also remained anonymous, which resulted in a lack of insight into their individual conditions. Also it’s possible that those who responded may have systematically differed from those who did not respond, related to our outcomes of interest. Thus, only acute responses were examined in the present study. Longer longitudinal follow-up is needed to examine the sub-acute and long-term psychological complications, such as posttraumatic stress disorder. We also do not know if any of the participants were infected with COVID-19, so we do not have an insight into their interconnectedness, which also needs to be examined. Future research with a longer post-COVID-19 observational period could help to advance our knowledge in this regard.

Postpartum women have an increased risk of NPMADs during the pandemic and the state of emergency police lockdown in Serbia. An elevation of risk of NPMADs was linked to several sociodemographic characteristics, quarantine and social isolation, as well as having emotional problems. Postpartum compared to non-postpartum women were more anxious and had feelings of helplessness, which resulted in an overestimation in the need for social support, during the social isolation. As a consequence of stigma during the postpartum period, women underestimated the need for psychological support. Only 1.9% of women reported having family support. Knowledge of the factors that increase the risk of NPMADs during the pandemic will help us mitigate or manage mental health disorders during pandemics.
